# Study on the Mechanism of Üstikuddus Sherbiti in Ischemic Cerebrovascular Diseases: Based on Network Pharmacology

**DOI:** 10.1155/2022/5581864

**Published:** 2022-04-08

**Authors:** Aman Gul, Mutalifu Aimaiti, Tuerhong Tuerxun, Raziye Amat, Ayinuer Reheman, Min Fang Zhang, Nassirhadjy Memtily

**Affiliations:** ^1^Central Laboratory, Xinjiang Medical University, Urumqi 830011, China; ^2^Department of Integrative Medicine, Huashan Hospital, Fudan University, Shanghai 200040, China; ^3^Institute of Integrated Traditional Chinese and Western Medicine, Fudan University, Shanghai 200040, China; ^4^Department of Neurology, The First Affiliated Hospital of Xinjiang Medical University, Urumqi 830054, China; ^5^Traditional Uyghur Medicine Institute, Xinjiang Medical University, Urumqi 830011, China

## Abstract

This paper aims to study the potential biological mechanism of Üstikuddus Sherbiti (ÜS) in the treatment of ischemic cerebrovascular diseases (ICVD) by the network pharmacology method. Traditional Chinese Medicine Systems Pharmacology (TCMSP) database was used to obtain effective constituents of ÜS by screening eligible oral utilization, drug similarity, and blood-brain barrier permeability threshold. By drug target prediction and stroke treatment target mining, 2 target data sets were analyzed to find intersection targets and the corresponding constituents were used as active constituents. An active constituent target network and an effective constituent target network were constructed by using Cytoscape 3.7.2 software. Degree parameters of the effective constituent target network were analyzed to find important effective constituents and targets. Through protein-protein interaction (PPI) analysis/Kyoto Encyclopedia of Genes and Genomes (KEGG) enrichment analysis, potential signaling pathways of ÜS in ischemic stroke were found out. AutoDock was used for molecular docking verification. A total of 90 active constituents of ÜS were screened out. There were 10 active constituents against ICVD, including quercetin, luteolin, kaempferol, and naringenin, and 10 important targets for anticerebral ischemia, namely, PIK3CA, APP, PIK3R1, MAPK1, MAPK3, AKT1, PRKCD, Fyn, RAC1, and NF-*κ*B1. Based on the protein interaction network, the important targets of ÜS were significantly enriched in PI3K-Akt signaling pathway, neuroactive ligand-receptor interaction pathway, Ras signaling pathway, etc. ÜS in ICVD has characteristics like multiple targets, multiple approaches, and multiple pathways. Results of molecular docking showed that the active components in ICVD had a good binding ability with the key targets. Its main biological mechanism may be related to the PI3K-Akt and Ras-MAPK centered signaling pathway. Our study demonstrated that ÜS exerted the effect of treating ICVD by regulating multiple targets and multiple channels with multiple components through the method of network pharmacology and molecular docking.

## 1. Introduction

Cerebral ischemia is one of the main causes of adult death and disability. Due to its high incidence, high disability, and high fatality rate, it has become one of the common problems faced by various countries in the field of public health [1, 2]. According to data released by the Chinese Center for Control and Prevention in 2017, the age-standardized prevalence of stroke was 873.4 per 100,000 people. Between 1985 and 2013, in the northern, central, and southern rural areas, the prevalence of stroke increased by 2.0 times, 1.5 times, and 1.2 times respectively. Overall, the prevalence of stroke is higher in rural areas than in urban areas. Although the mortality rate has shown a downward trend in recent years, there is still a large gap compared with developed countries. In China, this number is still increasing [3–5]. ICVD accounts for 60%–80% of strokes, including ischemic stroke and transient ischemic attack (TIA) [6]. According to the Organizational Standards for the Treatment of Acute Stroke 10172 (TOAST), ischemic stroke can be divided into large atherosclerosis, cardiac embolism, small artery occlusion, strokes of other definite etiology, and strokes of unknown etiology [7].

Thrombolysis and thrombectomy are conventional and effective treatments for ICVD. The treatment time window is short and is accompanied by reperfusion injury. Evidence-based medicine shows that intravenous thrombolysis and mechanical thrombolysis are aimed at the early pathological characteristics of ischemic stroke and are safe and effective treatment methods [8]. However, due to the short treatment time window (4.5 hours for intravenous thrombolysis and 16–24 hours for intravascular recanalization) and practical limitations of medical treatment, very few patients can be admitted to the hospital and receive treatment in the early stage of ischemia [9]. In some developed countries such as Europe and the United States, only 8%–10% of ischemic stroke patients have the opportunity to receive intravenous thrombolysis and mechanical thrombectomy [10], while in our country this figure is only 1.6% [2]. The complications of thrombolysis and ischemia-reperfusion injury cannot be ignored. Basic research shows that this injury may cause damage to the blood-brain barrier, excitatory amino acid toxicity, intracellular calcium overload, oxidative stress, cell apoptosis, and inflammation, etc.; destroy the homeostasis of the brain environment on the infarcted side; and aggravate the pathological damage of the brain tissue [11, 12].ÜS is composed of 12 kinds of single herbs, including the main herbs *Lavandula angustifolia* Mill., [13] *Adiantum flabellulatum* L., [14, 15] *Paeonia lactiflora* Pall., [16, 17] and *Anchusa Italica* Retz., [18–20] which play the role of unblocking a blocked blood vessel, relieving pain, and improving sleep. Complementary herbs include *Liquiritiae radix* [21, 22]*, Cordia dichotoma* seeds [23, 24], rose petals [25, 26], and raisins [27], to enhance the main herbs role. As the regulating herbs, *Althaea Rosea* L. [28] and *Viola tianschanica* Maxim. [29] adjust the properties and taste of the above herbs. As corrective medicine, celery seeds [30] and *Foeniculum vulgare* Mill. [31] correct the adverse effect of the above herbs on the stomach, as carminatives, digestives, galactogogues, and diuretics, and are used in treating gastrointestinal disorders. It is mainly used for the treatment of insomnia, depression, migraine, neurasthenia, and other diseases and auxiliary treatment of ischemic cardiovascular and cerebrovascular diseases [32]. In recent years, studies have found that ÜS single-medicine ingredients have strong anti-injury activity to the nervous system [18, 33, 34]. Modern studies have shown that ÜS's single-medicine ingredients are effective in treating many major diseases such as cardiovascular and cerebrovascular diseases, Alzheimer's, diabetes, and tumors and have neuroprotective effects on ICVD [32, 35], but its resistance to deficiency, the underlying mechanism of ICVD is still unclear. In recent years, some scholars have proposed to study the principles of prescriptions from the perspective of biological network regulation and understand the interaction between the complex chemical system of traditional medicine prescriptions and the complex biological systems of the body from the perspective of the network [36, 37].

With the help of the network pharmacology platform, this research explores the potential biology of ÜS in the treatment of ICVD from the perspective of systems biology by mining and analyzing the effective components and related targets of ÜS against the ICVD mechanism.

## 2. Materials and Methods

### 2.1. Screening of the Active Compounds in ÜS and Construction of the Compound-Target Network

The Traditional Chinese Medicine Systems Pharmacology (TCMSP) database and analysis platform is a network pharmacology database that integrates the information of traditional Chinese medicine ingredients and corresponding target information. The database contains key parameters for the absorption, distribution, metabolism, and excretion of traditional Chinese medicine components, including oral bioavailability (OB), drug-likeness (DL), blood-brain barrier permeability (BBB). In this study, we used the TCMSP platform and searched the PubChem database to collect all the components of ÜS and obtained the OB, DL, and BBB values of each component, with OB ≥ 30%, DL ≥ 0.18, and BBB ≥ -0.3 as candidate active ingredients basis.

### 2.2. Main Software and Database

- DrugBank database: https://www.drugbank.ca/
- Online Mendelian Inheritance in Man (OMIM) database: https://www.omim.org/
- Genetic Association Database (GAD): https://geneticassociationdb.nih.gov/
- Therapeutic Target Database (TTD): https://database.idrb.cqu.edu.cn/TTD/
- STITCH database: https://stitch.embl.de/
- SwissTargetPrediction database: https://www.swisstargetprediction.ch/
- DisGeNET database: https://www.disgenet.org/home/
- Venny 2.1 software: https://bioinfogp.cnb.csic.es/tools/venny/
- TCMIP database: https://www.tcmip.cn/TCMIP/index.php/Home/Index/index.html
- Google Scholar database: https://scholar.google.com.hk/?hl=zh-CN/
- UniProt database: https://www.uniprot.org
- DAVID database: https://david.ncifcrf.gov/
- AlzData database: https://www.alzdata.org/index.html
- OmicShare online data analysis platform: https://www.omicshare.com/tools/
- Cytoscape software: https://cytoscape.org/
- FunRich software: https://www.funrich.org/
- Metascape online data analysis platform: https://metascape.org/gp/index.html

### 2.3. Prediction of Target Points of Active Ingredients in ÜS

We use the TCMSP database to obtain the potential target information corresponding to each active ingredient screened in the ÜS, and search the targets from different sources in the UniProt database to obtain all target gene names, used for subsequent network pharmacology analysis.

### 2.4. Discovery of Targets for ICVD

We use the keywords “cerebral ischemia,” “ischemic stroke,” and “brain oxidative stress” to search DrugBank, OMIM, GAD, STITCH, and DisGeNET databases to obtain information on cerebral ischemia targets.

### 2.5. Core Target Screening

We enter the obtained targets of ICVD and the targets of ÜS components into the UniProt database to search for and obtain the gene symbols of all targets for subsequent analysis. In addition, we use Venny 2.1 online tool to draw the Venn diagram of the active ingredient targets of ÜS and the related targets of ICVD, and get the intersection target.

### 2.6. Construction and Topology Analysis of Component-Target Network

Cytoscape 3.7.2 software was used to construct a compound-target interaction network and an anticerebral ischemia drug effect component target interaction network. Traditional medicine components or targets are expressed as nodes in the network, and their connections are expressed as edges. Network analyzer in Cytoscape 3.7.2 software is used to calculate the degree of nodes (degree) parameters to evaluate the importance of medicinal ingredients and targets.

### 2.7. ÜS and the Distribution of Targets Related to ICVD in Brain Cells

The above-obtained intersection targets were input into the AlzData database [38] to analyze the anti-ICVD targets of ÜS in human brain neurons, endothelial cells, astrocytes, microglia, oligoprotruding cells, and oligodendrocytes. The expression of plasmonic precursor cells can be used to obtain the cell types in which these targets are highly expressed. Then Cytoscape software was used to construct a component-target-cell type network.

### 2.8. Construction of PPI Network and Screening of Topological Parameters

To understand the interaction of target proteins at the system level, we use the Metascape database to analyze and obtain a protein interaction network diagram.

### 2.9. Target Tissue Distribution and GO and KEGG Enrichment Analysis

We used the Database for Annotation, Visualization, and Integrated Discovery (DAVID) to perform Gene Ontology (GO) enrichment analysis and Kyoto Encyclopedia of Genes and Genomes (enrichment analysis of KEGG) signaling pathways to gain insight into the biological processes (BP), molecular functions (MF), and KEGG signaling pathways involved [39]. FunRich software was also used to perform tissue enrichment analysis on the target [40]. *P* < 0.01 was considered significant. We drew bubble charts by OmicShare tool, a free online platform for data analysis [41]. Moreover, DAVID was employed to analyze the pathway, and the R language ggplot2 package was used to draw the pathway enrichment analysis bubble chart. [42].

### 2.10. UHPLC-QE-MS Nontarget Metabolomics Analysis

The samples of ÜS were analyzed by an Agilent 1290 ultrahigh performance liquid chromatography (UHPLC) system with a Waters UPLC BEH C18 column (1.7 *μ*m 2.1 ∗ 100 mm). The column temperature was set at 55°C, and the sample injection volume was set at 5 *μ*L. The flow rate was set at 0.5 mL/min. The mobile phase consisted of 0.1% formic acid in water (A) and 0.1% formic acid in acetonitrile (B). The multistep linear elution gradient program was as follows: 0–11 min, 85–25% A; 11–12 min, 25–2% A; 12–14 min, 2–2% A; 14–14.1 min, 2–85% A; 14.1–15 min, 85–85% A; 15–16 min, 85–85% A. A *Q* Exactive Focus mass spectrometer coupled with Xcalibur software was employed to obtain the MS and MS/MS data based on the IDA acquisition mode. During each acquisition cycle, the mass range was from 100 to 1500, the top three of every cycle were screened, and the corresponding MS/MS data were further acquired. Sheath gas flow rate was 45 arb, aux gas flow rate 15 arb, capillary temperature 400°C, full MS resolution 70000, MS/MS resolution 17500, collision energy 15/30/45 in NCE mode, and spray voltage 4.0 kV (positive) or −3.6 kV (negative).

### 2.11. Molecular Docking Verification

To validate the compound-target associations, the AutoDock software (version 4.2) was used to perform the molecular docking program [43]. RCSB PDB (https://www.rcsb.org/) was used to retrieve and download the 3D structure files of key target proteins. 3D structure files of compounds were downloaded from PubChem (https://pubchem.ncbi.nlm.nih.gov/) [44]. Finally, the AutoDock platform was used for molecular docking verification. The binding energy was calculated to evaluate binding interactions between the compounds and their targets. Binding energy less than “−5” indicates a good binding interaction between the compound and target [45]. Ligand docking and binding site were visualized and analyzed with PyMOL and AutoDock Vina [46].

## 3. Results

### 3.1. Active Compounds in ÜS

We screened out 90 active ingredients based on OB ≥ 30%, DL ≥ 0.18, and BBB ≥ −0.3, including quercetin, luteolin, kaempferol, and naringenin. According to the OB value, Table 1 shows the top 10 OB active ingredients, including glycerol, isoliquiritigenin, shinpterocarpin, phaseol, ZINC519174, and formononetin.

### 3.2. Target Prediction of Active Ingredients of ÜS and Target Mining of ICVD

**Figure 1:**
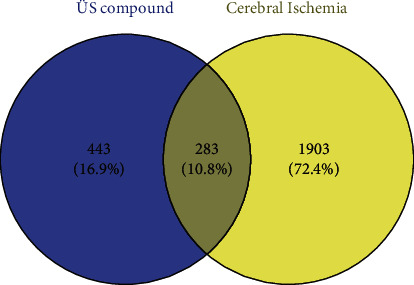
Venn plot of drug targets and ischemic stroke targets.

A total of 443 targets for the active ingredients of ÜS were obtained by searching the TCMSP database. Through keyword search of 5 online databases, DrugBank, OMIM, GAD, TTD, and DisGeNET, 1,903 related targets of ICVD were obtained. The 283 intersection targets obtained from the intersection of the above two data sets are used for subsequent network pharmacology analysis, as shown in Figure 1.

### 3.3. Construction and Analysis of the Active Ingredient-Target Interaction Network

Using Cytoscape 3.7.2 software to construct the active ingredient-target interaction network, a network with 343 points and 1994 edges was obtained. Among them, the top 10 targets ranked by degree are quercetin, luteolin, naringenin, jaranol, ZINC519174 (2R-7-Hydroxy-2-(4-hydroxyphenyl)-2,3-dihydrochromen-4-one), catechin, isoformononetin, kaempfride, isobavachin, and kaempferol as shown in Table 2.

### 3.4. Construction and Analysis of the Pharmacodynamic Ingredient-Target Interaction Network

**Figure 2:**
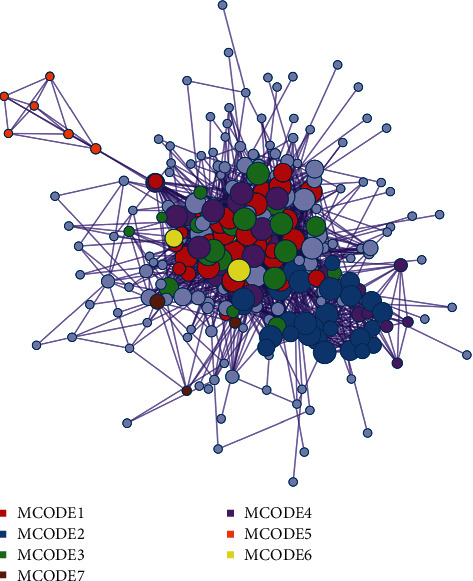
Protein-protein interaction network and MCODE components identified in the gene lists. MCODE_1: PI3K-Akt signaling pathway. MCODE_2: neuroactive ligand-receptor interaction. MCODE_3: pathways in cancer. MCODE_4: Rap1 signaling pathway. MCODE_5: functionalization of compounds, biological oxidation. MCODE_6: GO: regulation of transmembrane transporter activity. MCODE_7: dissolution of fibrin clot. PID UPA: UPAR pathway.

The obtained intersection target was taken as the pharmacodynamic target, and Cytoscape 3.7.2 was used to construct the pharmacodynamic ingredient-target interaction network (see Figure 2). The top 10 targets with degree values are shown in Table 2. The 10 important anti-ICVD targets are PIK3CA, APP, PIK3R1, MAPK1, MAPK3, AKT1, PRKCD, Fyn, RAC1, and NF-*κ*B1.

### 3.5. Analysis of Protein Interaction Relationship

**Figure 3:**
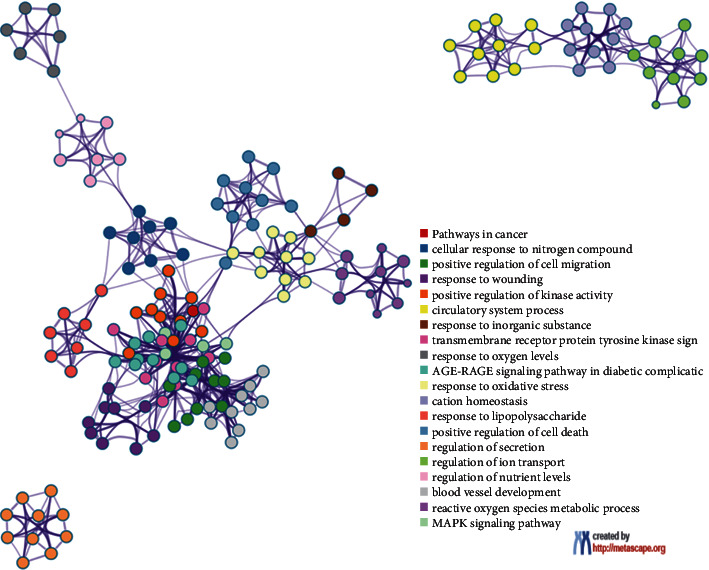
Compound-ischemic stroke-related target network of ÜS. Network of enriched terms: (a) colored by cluster ID, where nodes that share the same cluster ID are typically close to each other; (b) colored by *P* value, where terms containing more genes tend to have a more significant *P* value.

**Figure 4:**
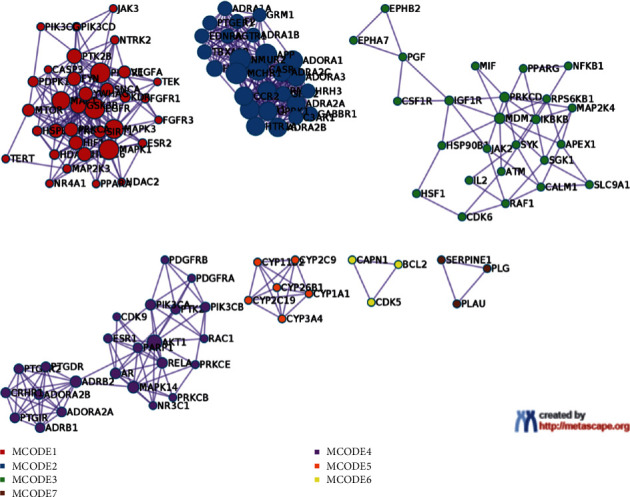
PPI network and MCODE components identified in the gene lists.

We import ÜS and anti-ischemic stroke-related targets into the Metascape database for analysis and obtain a protein interaction network diagram (see Figures 2–4). The results show that the interaction between these proteins is mainly manifested in the form of physical correlation, protein coexpression, pathways, and colocalization.

### 3.6. Organization of ÜS Target and GO Enrichment Analysis

**Figure 5:**
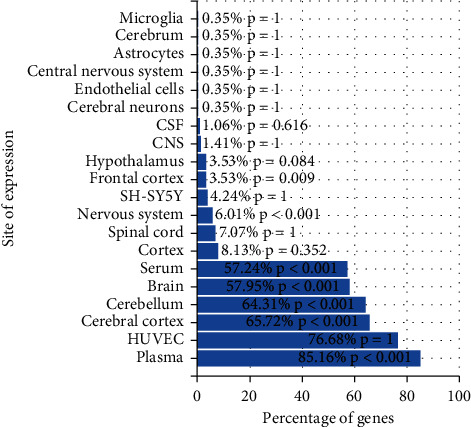
Target tissue distribution enrichment analysis.

**Figure 6:**
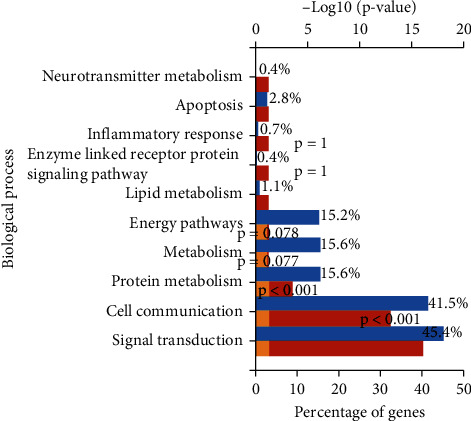
Enrichment analysis of biological processes.

**Figure 7:**
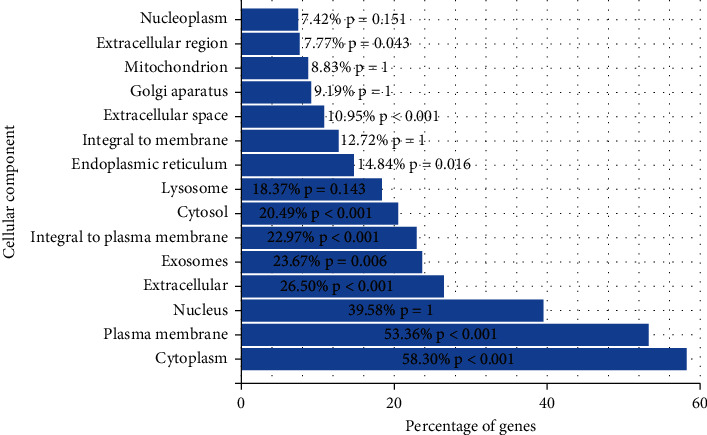
Cell component enrichment analysis.

**Figure 8:**
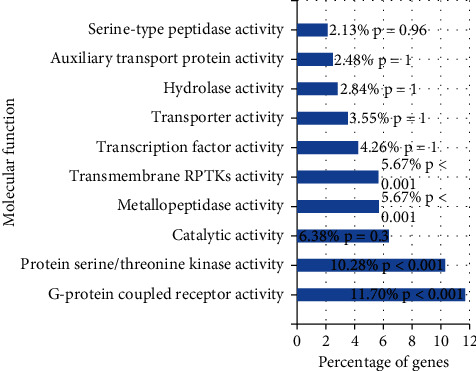
Molecular function enrichment analysis.

We used FunRich software to analyze the tissue distribution of ÜS targets (Figure 5). The results show that the targets of 90 active ingredients are significantly enriched in blood vessels (plasma) and human umbilical veins. Endothelial cells, cerebral cortex, cerebellum, nervous system, and other tissues and cells. We used DAVID to perform GO enrichment analysis for the ÜS target based on CC, BP, and MF terms. A total of 91 cell components, 780 biological processes, and 183 molecular functions were identified, as shown in Figure 6. It consists of 51 cell components, 387 biological processes, and 78 molecular functions with statistical significance (thresholds: count: 2, ease: 0.01). Sorted by *P* value, the smallest 15 cell components (Figure 7) were selected, and the biological processes (Figure 6) and molecular functions were displayed (Figure 8). We have noticed that some biological processes and molecular functions may be related to ICVD, such as response to hypoxia, inflammatory response, negative regulation of cell death, and calcium channel activity, indicating that the ÜS is effective for ischemic stroke and has potential therapeutic effects.

### 3.7. Analysis of Brain Cell Distribution of ÜS against ICVD Targets

**Figure 9:**
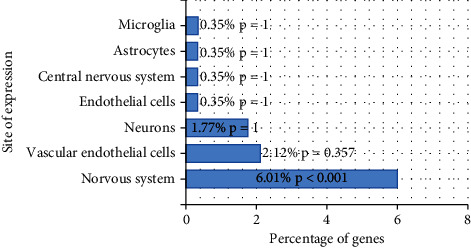
Target brain cell distribution enrichment analysis.

**Figure 10:**
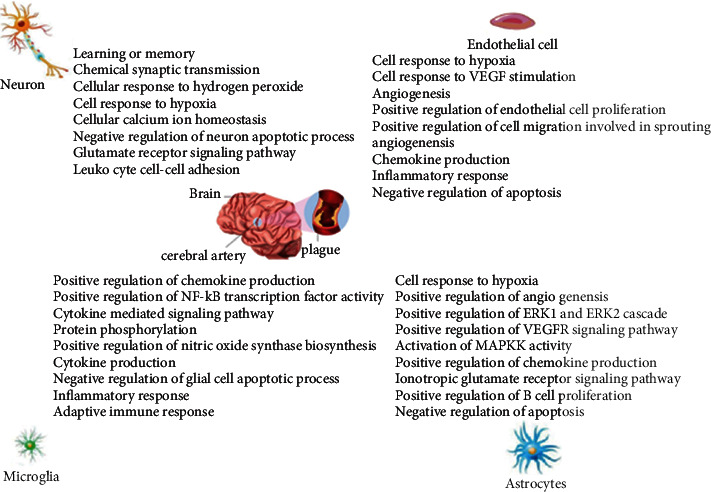
The biological process of ÜS in different cells of the neurovascular unit.

The Venn diagram of the component targets of ÜS and the disease targets of ICVD was drawn, and 283 overlapping targets were obtained. The 283 targets were entered into the AlzData database, and the expression of these targets in different cells was analyzed. Cytoscape 3.7.2 software was used to construct a component-anti-ICVD target-cell interaction network. The experimental results show (Figures 9 and 10) that the cell types are sorted by the number of targets, namely, neurons (112), endothelial cells (107), astrocytes (98), and microglia (72).

### 3.8. Analysis of KEGG Pathway, the Anti-ICVD Target

**Figure 11:**
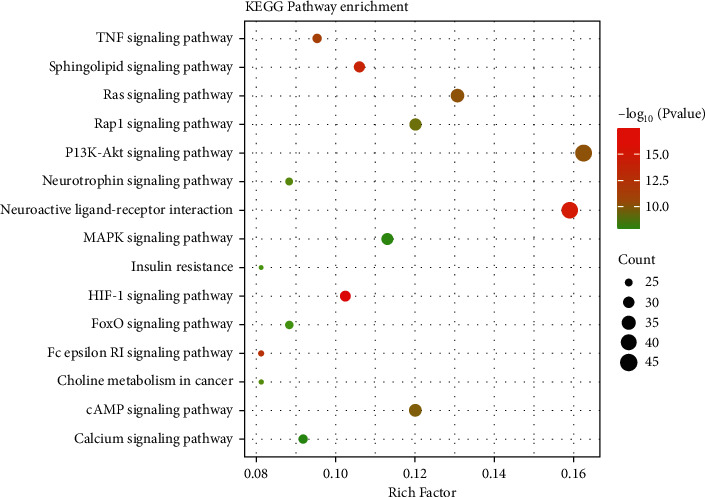
KEGG signaling pathway analysis of the core PPI network.

We use DAVID to perform KEGG enrichment analysis on the core PPI network. Sorted by *P* value, the top 15 signaling pathways related to ICVD are visualized (Figure 11), including pathways (Figures 2–4). The results show that the anti-ICVD effect of ÜS mainly involves PI3K-Akt signaling pathway, neuroactive ligand-receptor interaction, Ras signaling pathway, Rap1 signaling pathway, MAPK signaling pathway, and HIF-1 signaling pathway.

### 3.9. Components Analysis of ÜS

**Figure 12:**
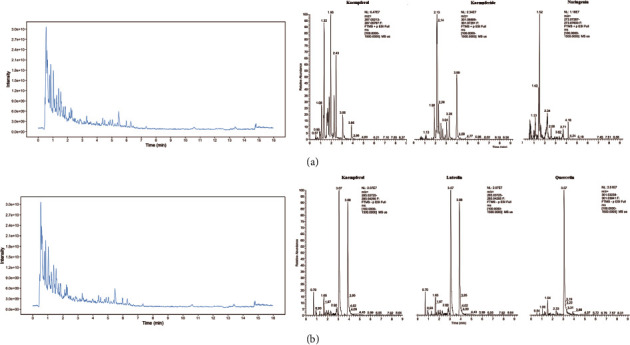
Identification of chemical components of ÜS. ÜS samples were examined by UHPLC-MS/MS. Total and main ion chromatography in positive (a) and negative (b) ion modes for ÜS samples are shown. UHPLC-MS/MS: ultrahigh performance liquid chromatography-tandem mass spectrometry.

To identify the, ÜS samples were analyzed by UHPLC-MS/MS. The total positive and some main major chemical components (Figure 12(a)), total and some main major chemical components negative (Figure 12(b)) ion chromatograms of ÜS demonstrated, in the chemical composition of all compounds. Several main components were demonstrated in ÜS, as shown in Figure 12(a) and 12(b), and 5 compounds were distinguished: (1) quercetin, (2) luteolin, (3) naringenin, (4) kaempfride, and (5) kaempferol.

### 3.10. Molecular Docking Verification

**Figure 13:**
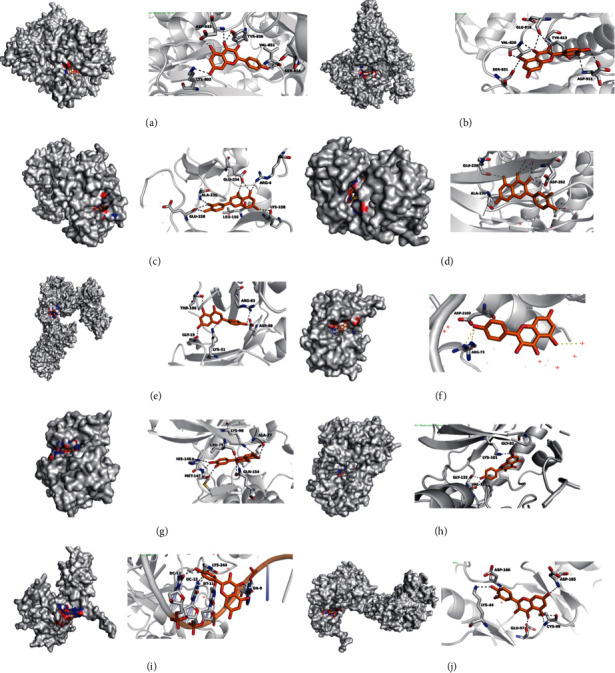
The conformations of some important compounds and key targets. Compound-target interactions with binding energy less than −5.0 kcal/mol are shown: (a) PIK3CA with kaempferol, (b) PIK3RI with quercetin, (c) AKT1 with luteolin, (d) AKT1I with quercetin, (e) MAPK1 with luteolin, (f) mTOR with kaempferol, (g) MEK1 with quercetin, (h) MEK2 with kaempferol, (i) NF-*κ*B1 with quercetin, and (j) IKK*β* with quercetin.

Compound-target interactions with binding energy less than −5.0 kcal/mol are shown in Table 3 and Figure 13, including PIK3CA with kaempferol, PIK3RI with quercetin, AKT1 with luteolin, AKT1 inhibitor with quercetin, MAPK1 with luteolin, mTOR with kaempferol, MEK1 with quercetin, MEK2 with kaempferol, NF-*κ*B1 with quercetin, and IKK*β* with quercetin.

### 3.11. Target Path Analysis

**Figure 14:**
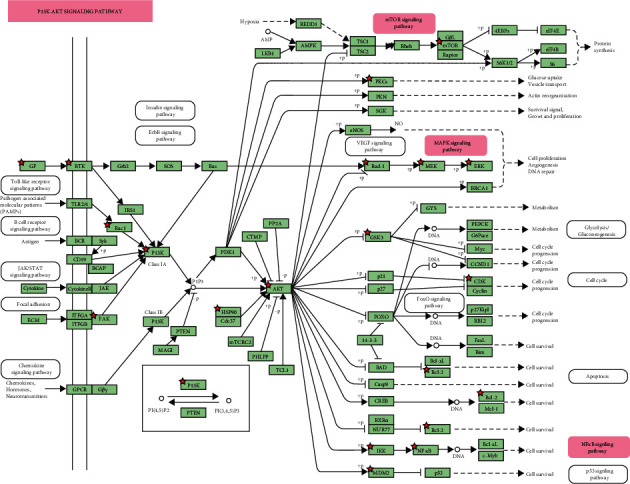
Pathway map of ÜS against ICVD. The key targets of ÜS in the treatment of ICVD are shown as rose in the PI3K/AKT signaling pathway.

The pathway map of ÜS in treating ICVD was obtained from KEGG PATHWAY Database, as shown in Figure 14. The related pathways were marked in red, and the targets of ÜS in treating ICVD were marked in rose. The results showed that the main pathways of ÜS in treating ICVD included TNF signaling pathway, MAPK signaling pathway, NF-*κ*B signaling pathway, and PI3K/AKT signaling pathway.

## 4. Discussion

More and more evidence shows that the pathophysiological mechanism of ICVD is very complicated, and a variety of biological processes and multiple signal pathways are involved in the process of ischemia-reperfusion injury. Traditional herb medicine is believed to be able to target energy metabolism and oxidation, multiple targets, multiple links, and multiple pathways such as stress, inflammation, endoplasmic reticulum stress, and apoptosis play a therapeutic role, which has obvious characteristics compared with traditional single compounds [47]. In addition, multitarget drugs avoid overregulation, with theoretically fewer side effects. Network pharmacology is from systems biology and biological networks. A research method to clarify the occurrence and development of diseases from two perspectives. Through the collection of traditional herbal medicine components and target points, an interaction network is constructed to further investigate the regulatory effect of traditional herbal medicine on different signals, thereby revealing the mechanism of traditional herbal medicine. This study explores the mechanism of ÜS in the treatment of ICVD is also based on the nonlinear interaction between their multiple components and the multiple targets of the body disease. It is mainly used to treat insomnia, depression, migraine, and neurasthenia. Numerous studies have reported the good curative effect of each herb of ÜS in the treatment of ICVD, with others being antioxidant, anti-inflammatory, and cardiovascular protection activities [13, 16, 18, 48–50].

To systematically explore the anti-ischemic brain of ÜS, the pharmacodynamic material basis and potential biological mechanism of vascular diseases, we use network pharmacology platform technology, through active ingredient screening, pharmacodynamic target prediction and analysis, protein interaction analysis, and gene annotation enrichment analysis, we have obtained 10 important effective ingredients, namely, quercetin, luteolin, naringenin, jaranol, ZINC519174, catechin, isoformononetin, kaempfride, isobavachin, and kaempferol. 10 important signaling pathways, namely, PI3K-Akt signaling pathway, neuroactive ligand-receptor interaction, Ras signaling pathway, cAMP signaling pathway, Rap1 signaling pathway, MAPK signaling pathway, sphingolipid signaling pathway, HIF-1 signaling pathway, and TNF signaling pathway.

We used FunRich software to perform tissue distribution enrichment analysis on all predicted targets of ÜS and found that these targets were widely enriched in the nervous system and circulatory system. The results of GO enrichment analysis showed that the target was significantly enriched in the items of biological processes or analytical functions related to cerebral ischemia, such as response to hypoxia, inflammatory response, calcium channel activity, negative regulation of the apoptotic process, and positive regulation of ERK1 and ERK2 cascade. The 283 targets obtained by the intersection of the Venn diagrams are not only the targets of the 90 components of ÜS but also the therapeutic targets of ICVD. Using AlzData, a database related to single-cell sequencing, we analyzed the high-expressing cell types of these 283 targets and found that the distribution of these targets in nerve cells is quite different. There are 112 targets highly expressed in neurons, 107 targets highly expressed in endothelial cells, 98 cells highly expressed in astrocytes, and 72 targets highly expressed in microglia. The above analysis shows that ÜS can act not only on neurons, but also on endothelial cells, astrocytes, and glial cells to resist ICVD. Because the brain is a steady-state organ integrating blood vessels, nerves, and immunity, and the nervous system is extremely sensitive to changes in cerebral blood flow, the pathophysiology of ICVD is extremely complicated. In the past two centuries, a large number of studies showed that the complexity of the interaction between the brain and its vascular system is unmatched by other organs [51]. In this context, the National Institute of Neurological Disorders and Stroke of the National Institutes of Health first proposed the concept of “neurovascular unit (NVU)” which aims to emphasize the unique relationship between brain cells and cerebral blood vessels [51]. This conceptual model has brought new opportunities for the development of therapeutic drugs for cerebral ischemia. In the past, the research and development of anticerebral ischemia drugs focused more on neuroprotective effects and rarely involved glial and vascular aspects. With the attention paid to the various cells that make up the neurovascular unit, the interaction between these cells has been emphasized; multitarget, multilink drugs have been developed, and a new era of comprehensive brain protection drug research and development has been opened. To understand the different effects of ÜS in different cells, we selected four types of cells, neurons, endothelial cells, astrocytes, and microglia, that constitute neurovascular units, and enriched for various types of cells with high expression. The biological process of the target. We found that ÜS plays different roles in different cells to fight cerebral ischemia. In neuronal cells, it mainly participates in antioxidative stress, antiapoptosis, antiexcitatory amino acid toxicity, anticalcium ion overload, etc.; promotes angiogenesis in endothelial cells; and inhibits inflammatory response, chemokine production, and other immune processes. Astrocytes regulate the hypoxic response of cells, resist oxidative stress, promote angiogenesis, regulate chemokines, resist excitatory amino acid toxicity, inhibit apoptosis, etc., participate in chemokines and cells in microglia, the signal pathways of factor production, nitric oxide synthase biosynthesis, anti-inflammatory response, and immune response may be related to NF-*κ*B and MAPK signal pathways.

The pharmacological effects of ÜS may not only involve predicted targets and ischemic targets in the database. To expand the target network and to explore and screen important target networks, we used the PPI theory to construct the PPI network of the ÜS target and the PPI network of ICVD. Convergence of complex networks, based on the theory of molecular interaction, combined with the view of complex systems, the status of nodes in biological networks is closely related to their biological importance. Interventions on key parts of the biological network can change the entire system and are a manifestation of network vulnerability [52]. We screened the topological parameters of the PPI network containing 1994 nodes (number of nodes: 343, number of edges: 1994) obtained from the intersection to find the more important node network from a system perspective, and analyzed the involvement of node proteins in the network-related signal pathways to explore the pharmacological mechanism of ÜS. This method has also been successfully applied to network pharmacology studies of other multitarget herbal medicines [53]. With the help of parameters including betweenness centrality (BC), degree centrality (DC), eigenvector centrality (EC), closeness centrality (CC), network centrality (NC), and local average connectivity (LAC), we got a 343-node, 1994-PPI network (string, number of nodes: 343, number of edges: 1994), which is defined as the core PPI network of ÜS against ICVD. The enrichment analysis of the KEGG signal pathway of the PPI network node obtained 15 important signal pathways related to cerebral ischemia. We focused on the PI3K/Akt signaling pathway, Ras signaling pathway, Rap1 signaling pathway, and MAPK signaling pathway, which are ranked high in the *P* value.

With research support, PI3K/Akt signaling pathway is one of the important therapeutic targets for ischemia-reperfusion injury. Upregulation of this signaling pathway helps to restore neurological function after ICVD, has antiapoptotic effects, inhibits autophagy and antioxidative stress, and promotes angiogenesis [3, 54–56]. Therefore, the PI3K/Akt signaling pathway may be one of the important signaling pathways for ÜS to treat cerebral ischemia and ICVD.

With research support, the Ras signaling pathway is one of the important therapeutic targets for ischemia-reperfusion injury [57]. Blocking the angiotensin II AT1 receptor in the cerebral microvasculature can prevent cerebral ischemia and inflammation, the presence and regulation of the local renin-angiotensin system (RAS) in the cerebral microvasculature of hypertension [57]. Plasma levels of AnxA1 are reduced in stroke, and AnxA1 can act on human platelets, thereby inhibiting the classic thrombin-induced internal-outside signaling events; for example, Akt activation, intracellular calcium release, and Ras-related protein 1 (Rap1) expression reduce *αβ* activation without changing its surface expression [58]. The MAPK signaling pathway is also one of the important therapeutic targets for ischemia-reperfusion injury [59]. Mitogen-activated protein kinase/extracellular signal-regulated kinase (MAPK/ERK), the pathway is inhibited in wild-type glial cells after brain IR injury but is reactivated in glial cells where the Mst1 gene is knocked out. Therefore, blocking the MAPK/ERK pathway can induce mitochondrial damage eliminates the beneficial effects of macrophage stimulator 1 (MST1) loss during brain IR injury [59]. HIF-1 and erythropoietin can be regulated by PI3K/Akt in brain tissue and affect brain deficiency, anti-inflammatory effect after blood reperfusion [60].

It is worth noting that among the top 10 important medicinal ingredients of ÜS against ICVD, quercetin [61], luteolin [62], naringenin [63], jaranol [64], catechin [65], isoformononetin [66], kaempfride [67], isobavachin [68], and kaempferol [69] have been proven to have neuroprotective effects, with the exception of ZINC519174. Quercetin can improve cell membrane integrity via decreasing lipid peroxidation. Apoptotic cell death is inhibited by quercetin via downregulation of Bax and caspases and upregulation of Bcl-2 [70]. Quercetin can modulate autophagy (inhibition/induction) in decreasing cerebral ischemia/reperfusion (I/R) injury [71]. Nanoparticles have been applied for the delivery of quercetin, enhancing its bioavailability and efficacy in the alleviation of I/R injury. Noteworthy, clinical trials have also confirmed the capability of quercetin in reducing I/R injury [72]. The neuroprotective effect of kaempferol has been confirmed in the ICVD [69, 73]. Kaempferol was responsible for significantly reducing the phosphorylation of NF-kB p65 in addition to inhibiting its translocation to the nucleus [69]. Luteolin protects against ICVD, potentially via regulation of the PI3K-Akt signaling, SIRT3, AMPK, and mTOR signaling pathway [62, 74]. The neuroprotective effect of naringenin is mediated through suppression of NF-*κ*B signaling pathway, preventing ischemic stroke damage via antiapoptotic and antioxidant effects [75, 76]. Kaempfride has neuroprotective effects through alleviating oxidative stress and enhancing the BDNF/TrkB/CREB pathway [77]. These findings suggested that quercetin, luteolin, naringenin, kaempfride, and kaempferol might be a promising choice for the intervention of ICVD.

PPI analysis showed that PIK3CA, APP, PIK3R1, MAPK1, MAPK3, AKT1, PRKCD, Fyn, RAC1, and NF-*κ*B1 were the top ten targets with high degrees. Followed by cluster of the PPI network, the network could be divided into four modules, which were mainly related to angiogenesis, inflammation, coagulation, and blood-brain barrier [78]. Accumulating evidence has confirmed that, under the injuries of stroke, cerebral ischemia, and I/R and activation of PI3K/Akt or PI3K/Akt/mTOR signaling pathway, PIK3CA, PIK3R1, AKT1, MAPK1, MAPK3 are main genes that play important roles in promoting cell survival and reducing cellular apoptosis [79, 80]. Strokes can trigger accelerated *β*-amyloid deposition, most likely through interference with amyloid clearance pathways [81]. PRKCD mediates cerebral reperfusion injury [82], and Fyn mediates PSD-95Y523 phosphorylation, which may be responsible for the excitotoxic signal cascades and neuronal apoptosis in the brain ischemia and A*β* neurotoxicity [83]. RAC1 regulation of Notch2 in mediating ICVD-induced production of injurious ROS and cell death in vitro and in vivo in the short term [84].

In our research, ÜS samples were examined by UHPLC-MS/MS. The main chemical components of ÜS, quercetin, luteolin, naringenin, kaempfride, and kaempferol, were examined. Compound-target interactions verified molecular docking, [85]; these results also suggest the anti-ICVD activity of ÜS, through multiple targets and multiple pathways, and its main biological mechanism may be related to the PI3K-Akt signaling pathway as the central signaling pathway.

PI3K-Akt signaling pathway, distributed in neuronal cells, endothelial cells, glial cells, immune cells, etc., can interact with a variety of ligands. The complex biological activity of this pathway is also reflected in its ability to bind ligands, the different activation of different signal transduction pathways in the cell, and triggering different downstream effects [54, 86]. The effect of ÜS on ICVD may be exerted through neurons, endothelial cells, glial cells, immune cells, etc.

## 5. Conclusions

In summary, the anti-ICVD effect of ÜS has the characteristics of multiple targets and multiple pathways, and its main biological mechanism may be related to the PI3K-Akt signaling pathway as the central signaling pathway. There are still some limitations in this study, such as the need to further optimize the methods for finding and confirming relevant targets of active ingredients, interaction of ÜS and neuroactive ligand-receptor, MAPK signaling, apoptosis, autophagy, and Rap1 signaling, and the relationship between these pathways requires experimental evidence. The rationality of the PI3K-Akt signaling pathway as the underlying biological basis for the treatment of different diseases in ÜS requires further biological evidence.

## Figures and Tables

**Table 1:** Top 10 OB active ingredients of ÜS.

	*Chemical name*	OB (%)	BBB	DL
1	Glycerol	90.78	−0.2	0.7
2	Shinpterocarpin	80.3	0.68	0.7
3	Phaseol	78.77	−0.1	0.6
4	Glyasperin F	75.84	−0.2	0.5
5	Inermine	75.18	0.4	0.5
6	Vestitol	74.66	0.3	0.2
7	Glyasperin M	72.67	0	0.6
8	ZINC519174	71.12	−0.3	0.2
9	Linalyl acetate	70.54	1.56	0.4
10	1-methoxyphaseollidin	69.98	0.48	0.6

*Note.* OB: oral bioavailability; DL: drug-likeness; BBB: blood-brain barrier permeability.

**Table 2:** Top 10 ÜS anti-ischemic stroke active ingredients and targets with degree.

Compound name	Probability	Degree	Component target	Degree
Quercetin	83	44	PIK3CA	108
Luteolin	53	46	APP	106
Naringenin	19	43	PIK3RI	106
Kaempfride	17	46	MAPK1	102
ZINC519174	17	45	MAPK3	86
Jaranol	13	46	AKT1	78
Catechin	13	43	PRKCD	68
Isoformononetin	13	39	Fyn	68
Isobavachin	11	44	RAC1	66
Kaempferol	11	13	NF-*κ*B1	64

**Table 3:** Docking score of the selected molecules for docking in the binding site of PIK3CA, PIK3RI, AKT1, MAPK1, MAPK3, MEK1, MEK2, NF-*κ*B1A, IKK*β*, and mTOR proteins.

Legend receptor docking score (ΔG, kcal/mol)											
Legend receptor	PIK3CA	PIK3RI	AKT1	AKT1I	MAPK1	MAPK3	mTOR	MEK1	MEK2	NF-*κ*B1A	IKK*β*
PDB ID	6OAC	5UBT	3OCB	3MVH	4CFE	4QTB	4DRI	3WIG	1S9I	1SVC	4KIK
Baicalein	−8.169	−7.94	−6.977	−5.591	−7.003	−6.274	−7.923	−9.682	−6.57	−3.33	−9.09
Kaempferide	−6.965	−8.251	−6.612	−7.074	−6.979	−6.127	−7.584	−8.611	−6.124		−10.012
Kaempferol	−11.174	−10.334	−6.179	−7.138	−6.995	−6.201	−8.101	−10.606	−10.038	−3.788	−10.044
Luteolin	−9.923	−11.827	−8.272	−6.495	−7.161	−6.323	−7.918	−11.405	−7.747	−5.29	−11.335
Naringenin	−8.491	−9.383	−6.571	−6.342	−7.316	−6.516	−8.047	−10.492	−7.82		−8.047
Quercetin	−10.365	−12.24	−7.74	−7.986	−6.849	−6.525	−7.237	−11.572	7.384	−5.684	−11.547

## Data Availability

The data used to support the findings of this study are available from the corresponding author upon request.
